# Far beyond the duality between truth and lies: meanings about fake news revealed by nursing

**DOI:** 10.1590/0034-7167-2025-0124

**Published:** 2026-08-21

**Authors:** Luana dos Santos Costa, Laura Johanson da Silva, Sabrina da Costa Machado Duarte, Thiago Privado da Silva, Carla Aparecida Arena Ventura, Ana Larissa Araujo Nogueira, Ítalo Rodolfo Silva

**Affiliations:** I Universidade Federal do Rio de Janeiro. Rio de Janeiro, Rio de Janeiro, Brazil; II Universidade Federal do Estado do Rio de Janeiro. Rio de Janeiro, Rio de Janeiro, Brazil; III Universidade de São Paulo. Ribeirão Preto, São Paulo, Brazil

**Keywords:** Nursing, Health, Disinformation, Nurse Practitioners, Knowledge Management., Enfermería, Salud, Infodemia, Desinformación, Enfermeras Practicantes, Gestión del Conocimiento.

## Abstract

**Objectives:**

to understand, from a complex perspective, the meanings of fake news in health and nursing as uncovered by nursing professionals.

**Methods:**

qualitative research, whose theoretical and methodological frameworks were, respectively, Complexity Theory and Grounded Theory. Participants were 25 nursing professionals from a public hospital and a family clinic in Rio de Janeiro. Semi-structured interviews were conducted between November 2021 and October 2023.

**Results:**

for nursing professionals, fake news is a complex phenomenon that presents form, content, and intentionality capable of projecting specific dynamics of production, propagation, and consumption of this information. The *modus operandi* of fake news finds gaps in social media control and algorithmic dynamics that favor its negative impact, including on science as the authority of discourse.

**Final Considerations:**

nursing professionals understand fake news as a phenomenon that transcends the duality between truth and falsehood, negatively impacting public health.

## RESUMEN

**Objetivos:**

comprender, desde una perspectiva compleja, el significado de las noticias falsas en salud y enfermería, según lo revelado por profesionales de enfermería.

**Métodos:**

investigación cualitativa, cuyos marcos teóricos y metodológicos fueron la Teoría de la Complejidad y la *Grounded Theory*. Participaron 25 profesionales de enfermería de un hospital público y una clínica de familia en Río de Janeiro. Se realizaron entrevistas semiestructuradas entre noviembre de 2021 y octubre de 2023.

**Resultados:**

las *fake news* son un fenómeno complejo que presenta forma, contenido e intencionalidad capaces de proyectar dinámicas específicas de producción, propagación y consumo de esta información. El *modus operandi* de las *fake news* encuentra lagunas en el control de las redes sociales y en la dinámica algorítmica que favorecen su impacto negativo, incluso sobre la ciencia como autoridad del discurso.

**Consideraciones Finales:**

los profesionales entienden las *fake news* como un fenómeno que trasciende la dualidad entre verdad y falsedad, impactando la salud pública.

## INTRODUCTION

In recent years, especially since the COVID-19 pandemic, across a wide range of social segments, we have come across discussions, causes, and effects related to fake news^(1)^. From a linear perspective, the expression could simply mean “false news”; however, due to the dynamics with which they are created, disseminated, and consumed, it is pertinent to understand fake news as a multifaceted phenomenon capable of intentionally or unintentionally influencing human behavior in certain decision-making processes that can compromise different sectors of society, from economic and political situations to those that affect health or directly violate human rights and dignity^(2,3)^.

As a complex phenomenon, it is important to emphasize that fake news does not conform to a reality limited to the time and space in which information and communication technologies associated with the advancement of the internet are experienced. Humanity has always been a product and producer of actions that generate, in the field of knowledge, distortions of reality, even if such actions were deliberately produced to explain reality itself through myths and allegories^(3,4)^, or even for political ends consecrated throughout history, as in the emblematic defamation trial of the Queen of France, Marie Antoinette, whose negative newspaper articles about the monarch intensified subjects’ feelings of revulsion towards King Louis XVI’s leadership and even towards the French monarchical regime^(5)^. In this case, we have one of the first historical records of what is understood today as fake news.

Currently, what makes the fake news phenomenon unique is not just the intentionality of generating fake news, but a set of factors that make it a multifaceted phenomenon, whose factors involve everything from the speed of information propagated virtually to difficulties related to the knowledge management process^(6)^, such as the need for proficiency and scientific literacy in society on the most diverse contents that are thematic targets of fake news, which includes the context of human health and well-being^(7)^. Furthermore, another element that deserves to be highlighted is the designation of what is now conceived as “post-truth”^(7,8)^.

Post-truth is a neologism that involves people’s tendency to construct a given common reality with significant expressiveness that is capable of influencing public opinion. In post-truth, the projection of reality occurs under the expressive guidance of emotions and personal and collective beliefs^(8)^ so that the objective facts of reality have less value in how people project their ways of conceiving the phenomena that surround them.

Therefore, it would be superficial to understand that fake news does not intentionally influence collective behavior, as is most intensely observed in contexts of political polarization, where emotional biases substantially influence the search for information that merely validates how certain groups of people understand reality. This situation extends to the healthcare context, as highlighted by questions about the efficacy of COVID-19 vaccines or even about unscientific therapeutic approaches for the disease resulting from the recent pandemic^(9)^.

From the above, behaviors that border on denialism in the health context can be inferred, which includes professionals themselves in the decision-making processes for clinical approaches that are far from the best scientific evidence^(10)^. Thus, fake news that establishes connections with the preconceived understanding of those who consume it can, of course, reinforce behaviors to which they were already inclined based on their own beliefs and values.

Given the implications of fake news that affect human behavior, nursing deserves attention as the largest professional category in the healthcare sector and, above all, for the role it plays in caring for patients and their families, especially in the context of popularizing scientific knowledge for health through health education initiatives. The sociopolitical role of nurses also centers on their ability to address the challenges that affect public health, which includes the potential links between fake news and the health-disease process^(11)^. Based on the above, the following research problem is raised: what meanings do nursing professionals reveal about fake news in health and nursing?

## OBJECTIVES

To understand, from a complex perspective, the meanings of fake news in health and nursing uncovered by nursing professionals.

## METHODS

### Ethical aspects

This research was approved by the Research Ethics Committee. Study participants were fully informed about the research’s nature and purpose, and signed an Informed Consent Form. Participants’ identities were protected by alphanumeric designations, as throughout the article they were identified as HN (Hospital Nurse), HNT (Hospital Nursing Technician), PCN (Primary Care Nurse), and PCNT (Primary Care Nursing Technician), followed by their respective interview numbers.

### Theoretical-methodological framework

Complexity Theory was used for the data interpretation process. This framework was chosen for its ability to articulate principles that value the multidimensionality of social phenomena of knowledge, thus highlighting elements such as the pathology of knowledge sustained by fragmentation of information, as well as disconnection between knowledge and objective reality^(12)^.

For the data analysis process, Grounded Theory (GT) was used, in its constructivist aspect of the “Corbinian” school, whose comparative approach, theoretical sampling, elaboration and integration of concepts result in three paradigmatic dimensions, namely: conditions, actions-interactions, and consequences^(13)^.

### Study design

This is qualitative and explanatory research that correlated subcategories around categories to explain the reality of the object investigated^(14)^. For the analytical reliability of this research, Equator guidelines were used, notably the COnsolidated criteria for REporting Qualitative research guide.

### Methodological procedures

Participants were characterized based on a form with professional information about their training and length of service. To familiarize them with the topic at hand, a thematic awareness-raising activity was conducted, based on the understanding that the meanings of a given phenomenon must emerge from the deepest reflections on the reality it signifies^(14)^. Therefore, before beginning the interview, the lead researcher provided two materials in article format containing information about the title, journal, authors, institutions, a structured abstract, and even a link. She then asked participants to review the materials for as long as they considered necessary. After this process, the researcher questioned participants about the veracity of the materials provided. It is worth noting that one of them contained completely false information and was provided in printed format, but after the dynamic, it was duly collected.

Semi-structured interviews were then conducted, with the following leading questions: what do you understand by fake news? How do you perceive this reality in the healthcare context? How do you perceive fake news in the nursing context? Based on the responses, circular questions were developed to facilitate comprehensive data collection. The interviews took place in individual meetings, in a private setting, within the study setting itself, at pre-arranged times that did not interfere with interviewees’ work activities. The interviews were digitally recorded (audio) and lasted an average of 30 minutes each. They were not repeated with the same participant. Analytical memos were used throughout data collection and analysis, a process that occurred simultaneously within the GT.

The memos enabled reflection on the development of concepts, aiding in understanding the data theoretical saturation, i.e., when a concept presents theoretical density supported by its respective subcategories. The theoretical saturation process was discussed among researchers before data collection was completed.

Data collection was conducted by a nurse researcher with expertise in the data collection method and the adopted methodological framework. There were no conflicts of interest related to study participants or research settings.

### Study setting

Data were collected in two settings: a federal university hospital and a family clinic located in the city of Rio de Janeiro, RJ. Hospital settings offers several clinical specialties and approximately 550 beds. The family clinic chosen for the study opened in December 2010. The unit has 14 family health teams and benefits 52,000 people, providing 100% family health coverage. The choice of two study settings was based on the hypothesis that context can influence the information access and dissemination dynamics, both with regard to nursing professionals and the profile of patients in each healthcare setting.

### Data source

Nurses and nursing technicians participated in the study, comprising two sample groups, respectively: Primary Health Care (family clinic) nursing professionals; and Tertiary Health Care (hospital) nursing professionals. Participants with at least one year of professional experience in the institution, providing direct patient care as a nurse or nursing technician, were included. In hospital settings, data were collected from professionals working in adult inpatient units (medical and surgical clinics). Participants on leave of absence or any type of leave were excluded. Participants were invited in person at the study site. No participant refused or withdrew.

### Data collection and organization

Data collection took place between November 2021 and October 2023. Interview data were analyzed using the GT coding stages: open, axial, and selective integration. In open coding, the data were segmented into distinct and rigorously examined parts, and comparisons were made to capture similarities between the initial data (preliminary codes)^(13)^.

### Data analysis

From the comparative analysis of preliminary codes, these were grouped, a process that allowed the delimitation of conceptual codes that were presented as an abstract representation of a fact, object, or action that the researcher perceives as significant. The grouping of conceptual codes by similarity gave rise to subcategories, and depending on the analytical density and connections between them, categories were delimited^(13)^. In this regard, axial coding requires that the analyst already has some subcategories constructed to relate them to their category, with a view to generating more complete explanations about the phenomenon investigated^(13)^. In the integration phase, the category is developed from the theoretical abstractions of its subcategories.

The subcategories were ordered based on the paradigmatic model, which considered three components: conditions, which concern factors that influence the development of the phenomenon; actions-interactions, which deal with strategies for developing and addressing the problem; and consequences, which signal potential reactions based on the strategies implemented.

## RESULTS

Twenty-five nursing professionals participated in the study, including 17 nurses, 11 from hospital settings and six from family clinics, as well as eight nursing technicians, six from hospital settings and two from family clinics. The mean age of the professionals working in hospital settings was 35, while in family clinics, the mean age was 28.

The mean length of service as nursing professionals in hospital settings was 16 years for nursing technicians and 11 years for nurses. For family clinic professionals, the mean professional experience was two years for nursing technicians and four years for nurses. All nurses had at least a graduate degree. Among the nursing technicians, only three had a graduate degree.

The following category emerged from the analytical process: “Fake news far beyond the duality between truth and falsehood: meanings uncovered by nursing professionals”. The category was structured based on the following subcategories: “Meanings uncovered by nursing professionals about fake news”, which, in the paradigmatic model, assumes the form of “conditions”; “Systematics of the *modus operandi* of fake news from nursing professionals’ perspective”, which addresses the actions-interactions within the paradigmatic model; and “Science in question: the perspective signaled by nursing professionals about fake news in the scientific context”, which reveals the consequences of the phenomenon under investigation, positioned within the paradigmatic model. This category presents the nonlinear logic of fake news from the perspective of meanings uncovered by nursing professionals.

It is important to note that the development of the category and its respective subcategories resulted from the process of grouping 197 preliminary codes and 12 conceptual codes. Therefore, the excerpts presented are merely illustrative of data contextualization.

### Meanings uncovered by nursing professionals about fake news

In line with its title, this subcategory reveals the meanings nurses and nursing technicians uncovered about fake news. In this context, it was possible to identify that study participants understand this reality in a systemic way, as they are able to contextualize the existence of fake news beyond the occurrence of isolated false information.

Although study participants considered fake news to be unproven information, they also realized that they were not limited to its meaning, as they understood that there is a process that involves not only the duality between truth and lies, since, in fake news, these characteristics are intentionally implied.

Thus, for nursing professionals, fake news constitutes a process of elaboration and dissemination of lies that assumes content (false information, partially or completely), form (mechanisms of elaboration and dissemination, with emphasis on the internet, especially through social media) and intentionality directed at achieving apparently defined objectives (convincing a group of people/population to make decisions and/or maintain behaviors).

To better understand this process, based on what the participants indicated, below are highlighted excerpts that demonstrate the dynamics of fake news development in form-content-intentionality.

Form: the way something is said, and this involves both the media through which the content is disseminated and the transmitters of information. The data, in this sense, highlights: the argument from authority (who says the information); easily accessible language for easier understanding (how the information is said; language); and where the information is said.

Who says the information:

*For instance, the medical profession is a class that is overvalued in our society, and when one of them says something about a certain subject to the patient, it becomes an indisputable fact, and this can become dangerous.* (HN4)

How they say:

*I realized that the fake news circulating about COVID-19 was always written in a simplistic way, so people understood it, you know?! So, people end up truly understanding it. Because they understand, and for an unknown disease, where very little was known, it ends up making sense to that person or, at the very least, it raises doubts to the point where they think it might be true and pass it on.* (HN3)

Where they say:

*Fake news is news that is spread incorrectly on social media.* (PCN14)

*These are news items that appear on social media without any proof.* (PCNT20)

*I understand that this is fake news, wrong or distorted or false news through social media.* (HNT12)

Content: although nonspecific, the content, according to participants, is created with real-life data (elements of truth) that are associated with false information so that the construction of fake news assumes characteristics that touch, in various ways, on the truth; however, it is an intentionally distorted reality.

*Oh, they’re made up! People take a little piece of information and distort it, about that subject, you know?! So, there’s not enough evidence to prove it.* (HN02)

*Fake news is all distorted information, a mixture of lies and truths* [...] *that comes with some truths mixed with lies.* (PCN13)

*Fake news is anything that isn’t true, or that’s partially true. There’s fake news. There’s something in there that might be true.* (HNT07)

*I think people just take a hook from some news story and inflate it. I’m not saying they make it up. Most of it is distorted in some way.* (HN5)

*I think people take a hook from some news story and inflate it. I won’t say they make it up; most of it is distorted in some way.* (HNT05)

*Fake news is false news, without any basis in fact, right?! That’s it, news shared without proof, without any basis.* (PCN17)

*People take a little piece of information and distort it, about that subject, you know. So, there’s not enough evidence to prove it.* (HN2)

Intentionality: distortion of information, as described above, is directly embedded in the process of creating fake news, but it is also intentional on the part of those who create it. For nursing professionals, this news is, therefore, intentionally created to not tell the truth. According to them, this reality is prone to defamation, slander, and distortion of reality.

The intentionality observed by participants is a complex situation, as it involves everything from personal inclinations, influences of political polarization, to economic interests.

*In vaccination now, as there are people who do not want to get vaccinated, they end up influencing others with information that is not true.* (HN1)

*For instance, recently regarding the vaccine, some people have been spreading information that contradicts its effectiveness and discouraging people from getting vaccinated. I think it’s also closely linked to politics.* (HN6)

*I understand fake news to be any news that is misleading, defamatory or slanderous about any fact.* (HN4)

*For instance, fake news is now circulating about our minimum wage. They’re saying hospitals won’t adhere to this new law. Everyone’s approach is different, and this creates both expectations and tension. There’s also a political issue, for instance, the government spreading false information for its own benefit.* (HN8)

However, it is worth highlighting that there were no specific meanings presented about fake news in relation to hospital and Primary Health Care contexts, nor among nurses and nursing technicians.

### Systematics of the *modus operandi* of fake news from nursing professionals’ perspective

This subcategory reveals potential conditions for the success of fake news, according to nursing professionals. For them, the operationalization of fake news occurs primarily through social media as dissemination vehicles, as they directly influence the dynamics of sharing and consumption of this news, both through speed and algorithms, which maintain the thinking and behavior of polarized groups. This is a feedback loop of dissemination and consumption of fake news that influences: the mass spread of reports; the gullibility of people (patients) regarding what they consume in the media; and the accessibility and dissemination of information associated with polarized groups.

*So, I see some information circulating online. Some patients believe it, and then start following it. Even when there was no vaccine, there were people who didn’t believe in the disease.* (HN1)

*I understand that this is fake news, wrong or distorted or false news through social media: Facebook^®^, Instagram^®^, WhatsApp^®^.* (HN12)

*This distorted news usually comes from social media: Facebook^®^ and WhatsApp^®^. This makes it easier for us to encounter it because everyone nowadays has access to these media outlets.* (PCN15)

*I think the ease of access to social media greatly contributes to the spread of fake news. Nowadays, everyone has access to the internet and communicates with multiple people at the same time.* (HN23)

*The internet greatly facilitates our access to communication. We can find out what’s happening elsewhere in the world with just a click.* (HNT03)

*These are mass-circulated news stories, mainly through social media and apps like WhatsApp^®^* [...] *people stay in their little groups and share information they believe. It’s not always true, but no one in the group questions it.* (HN9)

Participants understand the amplification and spread of fake news based on the speed with which information is disseminated due to the agility afforded by the internet, as well as the ability to simultaneously reach multiple audiences. This amplification is also facilitated by the absence or limitations of strategies that act as barriers to monitoring/preventing the spread of fake news on social media.

*Ah, fake news is false news that’s posted on the internet, right? And then it takes on gigantic proportions.* (HN3)

*Social media is a platform where you can send messages to multiple people. On WhatsApp^®^, you receive these false news stories daily. Through it, you can spread the message very quickly.* (HNT22)

*I believe that the ease of access to these means, the lack of barriers and the speed with which you can disseminate the message facilitates dissemination of these messages.* (HN21)

*Recently, fake news has gained traction thanks to WhatsApp^®^, right?! Messages arrive so quickly. Sometimes, they haven’t even made it into the newspaper yet, but they’re already in groups on the social network.* (PCNT20)

The continuous and incessant nature of social media expands through the interactivity these media enable (algorithmic dynamics) to maintain audiences. For nursing professionals, the excess of information disseminated on social media also facilitates the spread of misleading news. Therefore, volume, frequency, and speed are factors perceived by research participants in the dynamics of fake news dissemination.

*On social media, information is daily, constant, 24 hours a day, posted all the time, and everyone can interact with that information.* (HN23)

*On the internet and social media, information comes in a whirlwind, all the time, all at once. It’s an avalanche of information all at once. And while you receive it, you can share it in a second.* (PCN18)

When considering the complexities involved in the development of fake news (form, content, intentionality, and the nature of the media used to disseminate it), participants revealed connections between fake news and the healthcare context, particularly when they realized that patients’ consumption and sharing of this news can increase vulnerabilities due to their lack of knowledge regarding several issues related to the health-disease process, among other related topics. Therefore, according to nursing professionals, this reality is linked to the population’s level of formal basic education and health literacy.

*I think the public lacks knowledge. Unfortunately, we have a population that isn’t very educated, that doesn’t understand the health-disease process, and they believe everything they see on the internet and end up spreading it.* (HNT03)

*I believe it’s largely due to a lack of knowledge. Patients are generally laypeople and don’t know much about health.* (HN01)

*Our population is very low-income, unaware of healthcare, but at the same time, everyone has WhatsApp^®^, where fake news abounds.* (PCN17)

In addition to formal knowledge about health, belief and value systems can influence the dynamics of consumption and dissemination of fake news among the population, according to the nursing professionals interviewed.

*I think it could be something like this: besides people not really seeking the veracity of the information, I think it could also be due to a lack of depth, perhaps knowledge, or even the willpower to verify whether the information is real or not. I believe it’s more because of this. People don’t want to actually stop and investigate.* (PCNT16)

*In the case of the vaccine, we’ve seen a major breakthrough in combating diseases. Millions of dollars have been invested. Many people, including scientists, have studied this, for the vaccine to be released. And many people, unfortunately, don’t believe it, even in the 21st century, even with proof that the vaccine works, and there are still people who believe it doesn’t work.* (HN02)

*Elderly people already have their own beliefs. It’s difficult to demystify everything they’ve believed for years. So, they’re resistant to change.* (HN7)

*Here, we have very humble users, not very literate, and many times they bring with them beliefs or information that neighbors or relatives pass on to them.* (HN9)

### Science in question: the perspective signaled by nursing professionals about fake news in the scientific context

The data for this subcategory were significantly informed by the awareness-raising process conducted with research participants, during which the researcher presented a fake news story and a true news story, as described in the study methodology. When asked how they perceive fake news in healthcare and nursing, participants indicated that they understand that this news also reaches the scientific context in the same dynamic (form, content, and intention) that they perceive in everyday life.

For nursing professionals, science and scientists represent an argument of authority over what they publish, and this socially ingrained representation influences the credibility of such news. However, the data revealed the importance of professionals’ scientific proficiency in identifying fake news within scientific contexts, as demonstrated by the following excerpts:

*These are messages that are circulated through social media or even in a fake article.* (HN10)

*It is news disguised as true, like, for instance, an article* [...] *because it is a scientific article, we are led to believe it, but it was published without having undergone validation.* (HN6)

*So, the information - some of it may not be true, but another part of it is* [...] *if you come to me with a very convincing speech, maybe you’ll end up convincing me because she went to college, then I’ll believe it.* (HN7)

*Fake news is when information circulates on social media, or an article considered scientific is published with false information and is propagated on the network.* (PCN18)

*Vaccination coverage has dropped significantly since the pandemic, and I think that, since the 90s, it started with a very large anti-vaccine movement, including because of a poorly written article published in a famous magazine and everything.* (HN19)

*There was a highly regarded journal that published an important article. I think it was about COVID-19. Later, they realized the article wasn’t true. So, the journal itself had to issue a statement that the article wasn’t true. But it’s very difficult for us, an important journal, to pick it up and read an article and say, “I don’t think that’s true!”.* (HN23)

*When it’s a scientific article, it’s already proven, more serious. The person who wrote it took the time to study it, and I believe it’s truthful* [...] *checking to see if other people are talking about the same subject elsewhere. Don’t just rely on what one person says.* (HN21)

Study participants reinforced the importance of understanding fake news as a multifaceted phenomenon, whose production and dissemination dynamics go beyond the simple identification of fake news or a common-sense context, also reaching the scientific arena. These representations imply that fake news has the appropriate argumentation authority (supposedly scientific) and argumentation authority (because the scientist is the one reporting the news).

## DISCUSSION

The complexity involved in the development of fake news is related to the behavior of access, consumption, and dissemination of false information, whether constructed intentionally or not^(15)^. In this context, the pathology of knowledge, as designated in Complexity Theory, by presenting the decontextualization of knowledge^(13)^, assumes, in the context of fake news, the objective of serving purposes of collective manipulation, or simply because it is information that becomes obsolete over time, as occurs with outdated research results.

Such complexities, associated with the lack of regulatory policies for the flow of information on the internet, political-partisan polarizations and the weaknesses of knowledge for critical thinking that establishes dialectical possibilities for confronting reality through resources and strategies that allow for qualifying criteria of truth, can foster deregulation between dissemination of fake news^(1)^ and, consequently, devaluation of scientific knowledge.

From a linear perspective, the understanding of fake news might suggest a free translation of the expression when it comes to Brazilian Portuguese, signaling a reductionist understanding of “false news”. By doing so, one could infer that participants view fake news as news reported by an individual telling a story, the interpretation of which is at the discretion of the transmitter. However, the literature reiterates that the production of fake news can be carried out consciously or unconsciously, based on belief systems, values, or even cognitive aspects^(2,7)^.

The nursing professionals in this study understand fake news as a multifaceted phenomenon, not just polysemic. In this context, fake news is understood as deliberately false or misleading information, with intentionality, form, and dynamics that find their way into gaps related to a lack of scientific or journalistic evidence based on news stories published online. Therefore, the understanding is reiterated that this topic should not be analyzed through a reductionist lens^(16)^. However, the expansion of the meanings of fake news supports the understanding that proposes to encompass not only deliberately false texts published on social media, but also shared videos and images^(17)^.

It is worth noting, however, that according to the nursing professionals surveyed, this phenomenon is concentrated in the internet, especially on social media. This data is supported by the fact that fake news essentially involves manipulating news stories to appear real and the viral power they establish based on the way they are created, as well as their dissemination online through social media^(18)^.

The internet and social media are easily accessible means of communication, and information consumption has increased exponentially in recent years^(2)^. Furthermore, social media represents society in the digital world, a context where individuals connect through the internet, fostering communication, but not always for pluralistic thinking, as they are influenced by algorithms that favor the maintenance of like-minded groups.

On these platforms, anyone, regardless of their credibility, is allowed to disseminate information with its power of propagation^(2,6)^. Thus, the reality involved in the creation and propagation of fake news must reach the necessary alert for the use of social media, given the capacity of this information to dangerously influence public behavior and opinion, whose objective of deception, especially in the political sphere, is to convince the reader, for instance, to determine positions of control under a disruptive condition of reality, based on disputes of narratives^(19)^.

The power of fake news to spread on social media, according to the participants in this study, lies in the speed and frequency with which this information is transmitted. This is due, among other factors, to the rapid development of Artificial Intelligence (AI), which, through algorithms, has the ability to triangulate data that allows the maintenance of specific groups of people for ideas and projections of common beliefs^(20)^. Thus, AI acts to influence message exchanges, suggesting new connections and consumption of information designed for greater interactivity with specific social groups and the like.

Algorithms are a technical and abstract grouping introduced into the information system, with the aim of interceding in the relationships and news we receive^(21)^, so this mechanism can pose risks to users’ choices and decision-making processes. Furthermore, algorithms are a set of mathematical codes designed to demonstrate a personal position by defining the *modus operandi* of information circulating on social media platforms^(22)^. Therefore, AI involved in social media can interfere with people’s sense of freedom and trust^(23)^. Added to the problem in question is the fact that social media platforms, in principle, are not concerned with the truth, but with the utility in which they seek to know and control information from a perspective of consumption and power^(24)^.

Another factor highlighted by participants in this study concerns societal misinformation about healthcare, and this reality is cited as a favorable context for the spread of fake news. Thus, throughout human history, misinformation has always been present in the construction of societies, under a logic of feedback for different purposes. However, what makes misinformation a growing reality in contemporary society is the ease of connections due to an extremely virtualized community^(25)^, coupled with the lack of critical thinking capable of questioning the information accessed.

Within a complex framework, theorist Morin^(13)^ emphasizes that the development of knowledge consists, based on language and thought, of a translation or reconstruction of knowledge in which the worldview, subjectivity, and principles of individuality are perpetuated. This paradigm highlights the importance of recognizing the potential fragmentation between mind and body, and emotion and reason, developing new concepts. Therefore, when conceptualizing fake news under the paradigm of complexity, it is understood that it constitutes a hybrid, plural ecosystem, fed by social heterogeneity through interpretative and dialogical narratives belonging to a given context where individuals act and think according to their social paradigm, which, in a way, converges with the concept of post-truth.

In post-truth, dissemination of false information vulgarizes lies and relativizes truth. The importance and credibility of traditional media outlets are diminished by personal judgments. Facts, meanwhile, take a back seat, so that, in the Postmodern era, truths, even scientific ones, are considered uncertain, while postmodern society is constantly being constructed daily through social media, with a high value placed on criticism, thus giving rise to fake news.

Fake news can be understood based on the meanings presented by the participants in this research, through the retroactive principle of complexity^(13)^, whose dynamics break the linear form of knowledge, placing it on another level, in which the producer and the product have autonomy in the information process, from the production to dissemination of the news. In this same logic, based on the principle of self-organization^(13)^, it can be understood that false information has autonomy in postmodern society, whose dependence on social media is present at the same time that information bubbles emerge and strengthen.

The data from this survey indicated that nursing professionals have difficulty identifying fake news simply because it was presented in the format of a scientific article. This situation may endorse the understanding that, even with criteria in place, fake news can still go unnoticed by the editorial process and be published under the guise of scientific authority. However, when fake news is identified in a supposedly scientific publication, it calls into question not only the media outlet and actors involved in its dissemination, but also science itself, in line with denialist thinking^(26)^. Furthermore, just like the complex principle of the ecology of action, once inserted into a system of dynamic and complex interactions, a given action can no longer be controlled, and its consequences become uncertain, beyond the reach of its causative agent^(12)^. Thus, the production of fake news disguised as a scientific article can have unimaginable consequences in the decision-making processes of all those who consider the information false, without question, to guide clinical and/or care conduct.

In addition to this, predatory journals, lacking a commitment to scientific integrity, can contribute to dissemination of false information in scientific article format. These media outlets violate the ethical integrity of science by adopting data fabrication, plagiarism, and falsification practices, while dispensing with peer review, among other important scientific integrity criteria^(26,27)^.

In this context, predatory journals publish articles offering a fast-publishing process with generalist titles. The problem with the growth of these predatory publications is primarily due to academic productivism, which prioritizes quantity over quality, aiming to feed back into the publishing industry, which is not committed to ethical and scientific integrity. This undermines the work of researchers, as well as the reliability and credibility of scientific information dissemination^(28)^.

### Study limitations

The limitations of the research are related to the epistemic field, since the nature of the participants inserted only in the public health context can, for instance, shape specific meanings about fake news in health and nursing.

### Contributions to nursing, health or public policy

By establishing direct possibilities for influencing human behavior, especially in relation to health, fake news constitutes an important subject of interest for public health. Thus, the meanings that nursing professionals reveal about this phenomenon in this study can significantly contribute to understanding the conditions, actions, interactions, and consequences of fake news in health and nursing. This can, for instance, allow for the understanding of the dynamics and *modus operandi* of this news, as well as strategies to address it, including in the context of science, healthcare, and education.

## FINAL CONSIDERATIONS

Nursing professionals understand fake news systematically, recognizing that it goes beyond the simple duality between truth and falsehood. For these professionals, fake news involves intentionality to influence behaviors or decisions. Furthermore, they understand that the construction of fake news occurs through a combination of form, content, and intentionality, with authoritative arguments and accessible language playing crucial roles in the dissemination and acceptance of this information.

The complexity involved in fake news, for nursing professionals, therefore lies in the axiomatic dynamics with which these stories are created, disseminated, consumed, and fed back. Thus, from the healthcare context to the production and consumption of science, fake news can influence dangerous behaviors, which jeopardize not only the quality of nursing and healthcare professionals’ work, but, most importantly, public health.

## Data Availability

The research data are available within the article.
