# Supplementary material for: Far beyond the duality between truth and lies: meanings about fake news revealed by nursing
Source: Rev Bras Enferm. 2026 Aug 21;79:e20250124. doi: 10.1590/0034-7167-2025-0124 (PMC13505563; doi:10.1590/0034-7167-2025-0124)
Supplement: Translation

# Translation: Muito além da dualidade entre verdade e mentira: esignificados sobre *fake news* desvelados pela enfermagem

**DOI:** 10.1590/0034-7167-2025-0124pt

**Authors:** Luana dos Santos Costa, Laura Johanson da Silva, Sabrina da Costa Machado Duarte, Thiago Privado da Silva, Carla Aparecida Arena Ventura, Ana Larissa Araujo Nogueira, Ítalo Rodolfo Silva

**Affiliations:** I Universidade Federal do Rio de Janeiro. Rio de Janeiro, Rio de Janeiro, Brasil; II Universidade Federal do Estado do Rio de Janeiro. Rio de Janeiro, Rio de Janeiro, Brasil; III Universidade de São Paulo. Ribeirão Preto, São Paulo, Brasil

**Keywords:** Enfermagem, Saúde, Desinformação, Profissionais de Enfermagem, Gestão do Conhecimento.

## Abstract

**Objetivos:**

compreender, sob a perspectiva complexa, significados sobre *fake news* na saúde e enfermagem desvelados por profissionais de enfermagem.

**Métodos:**

pesquisa qualitativa, cujos referencias teórico e metodológico foram, respectivamente, a Teoria da Complexidade e a *Grounded Theory*. Participaram 25 profissionais de enfermagem de um hospital público e de uma clínica da família do Rio de Janeiro. Foram empregadas entrevistas semiestruturadas entre novembro de 2021 e outubro de 2023.

**Resultados:**

para os profissionais de enfermagem, *fake news* é fenômeno complexo que apresenta forma, conteúdo e intencionalidade capazes de projetar dinâmicas específicas de produção, propagação e consumo dessas informações. O *modus operandi* das *fake news* encontra nas mídias sociais lacunas de controle e dinâmica algorítmica que favorecem o seu impacto negativo, inclusive na ciência como autoridade do discurso.

**Considerações Finais:**

os profissionais de enfermagem significam *fake news* como fenômeno que transcende a dualidade entre verdade e mentira, impactando negativamente a saúde pública.

## INTRODUÇÃO

Nos últimos anos, sobretudo a partir da pandemia de COVID-19, nos mais diferentes segmentos sociais, temos nos deparado com discussões, causas e efeitos relacionados às *fake news* ^(1)^. Sob uma perspectiva linear, a expressão, em sua tradução livre, poderia significar apenas o termo “notícia falsa”; porém, devido à dinâmica com que são elaboradas, difundidas e consumidas, faz-se pertinente compreender *fake news* como fenômeno multifacetado capaz de influenciar intencionalmente ou não comportamentos humanos para determinados processos decisórios que podem comprometer diferentes setores da sociedade, desde conjunturas econômicas, políticas e até mesmo aquelas que afetam a saúde ou que violam diretamente direitos e a dignidade da pessoa humana^(2,3)^.

Como fenômeno complexo, há que se destacar que *fake news* não conformam uma realidade que se limita ao tempo e espaço em que se vivenciam as tecnologias de informação e comunicação associadas aos progressos da internet. A humanidade sempre foi produto e produtora de ações que geram, no campo do conhecimento, distorções da realidade, ainda que deliberadamente tais ações fossem produzidas para explicar a própria realidade a partir de mitos e alegorias^(3,4)^ ou até mesmo para fins políticos consagrados no decurso da história, como no emblemático processo de difamação da Rainha da França, Maria Antonieta, cujos folhetins negativos sobre a monarca intensificaram o sentimento de repulsa dos súditos sobre a liderança do Rei Luís XVI e até mesmo sobre o regime monárquico francês^(5)^. Tem-se, nesse caso, um dos primeiros registros históricos do que se compreende, hoje, como *fake news*.

Atualmente, o que faz do fenômeno *fake news* singular não é apenas a intencionalidade de se gerar *fake news*, mas um conjunto de fatores que faz desse um fenômeno multifacetado, cujos fatores envolvem, desde a celeridade das informações propagadas virtualmente até as dificuldades relacionadas ao processo de gestão do conhecimento^(6)^, como a necessidade de proficiência e letramento científico da sociedade sobre os mais diferentes conteúdos que são alvos temáticos de *fake news*, o que inclui o contexto da saúde e bem-estar humano^(7)^. Ademais, outro elemento que merece destaque está na designação do que passa a ser concebido como “pós-verdade”^(7,8)^.

Pós-verdade é neologismo que envolve a inclinação das pessoas para construção de uma dada realidade comum com significativa expressividade que seja capaz de influenciar a opinião pública. Na pós-verdade, a projeção da realidade se dá sob a condução expressiva das emoções, crenças pessoais e coletivas^(8)^, de modo que os fatos objetivos da realidade têm menos valor para a forma como as pessoas projetam seus modos de conceber os fenômenos que as circundam.

Portanto, seria de um pensamento superficial o entendimento de que *fake news* não assumem uma projeção intencional de influenciar comportamentos coletivos, conforme se observa com mais intensidade em contextos de polarização política, cujas inclinações no campo das emoções influenciam substancialmente a busca por informações que apenas validam a forma como determinados grupos de pessoas compreendem a realidade. Essa conjuntura alcança o contexto da saúde, conforme pôde ser enfatizado a partir dos questionamentos sobre a eficácia das vacinas contra COVID-19 ou mesmo sobre condutas terapêuticas sem fundamentações científicas para a doença resultante da recente pandemia^(9)^.

A partir do exposto, depreendem-se comportamentos que tangenciam o negacionismo no contexto da saúde, o que inclui os próprios profissionais nos processos de tomada de decisão para as abordagens clínicas distanciadas das melhores evidências científicas^(10)^. Desse modo, *fake news* que estabelecem nexos com o entendimento preconcebido daqueles que a consomem podem, por suposto, reforçar condutas das quais já estavam inclinados sob sustentação de suas próprias crenças e valores.

Diante das implicações das *fake news* que afetam comportamentos humanos, a enfermagem merece atenção por ser a maior categoria profissional no setor saúde e, sobretudo, pelo papel que desempenha nos cuidados aos pacientes e seus familiares, especialmente no âmbito da popularização do conhecimento científico para a saúde a partir das ações relacionadas à educação em saúde. O papel sociopolítico do enfermeiro também está centrado em sua capacidade para o enfrentamento dos desafios que afetam a saúde pública, o que inclui as potenciais relações entre *fake news* e processo saúde-doença^(11)^. Pelo exposto, tem-se como problema de pesquisa a seguinte questão: quais os significados os profissionais de enfermagem desvelam sobre *fake news* na saúde e enfermagem?

## OBJETIVOS

Compreender, sob a perspectiva complexa, significados sobre *fake news* na saúde e enfermagem desvelados por profissionais de enfermagem.

## MÉTODOS

### Aspectos éticos

Esta pesquisa foi aprovada pelo Comitê de Ética em Pesquisa com Seres Humanos. Os participantes do estudo foram devidamente esclarecidos quanto à natureza e finalidade da pesquisa, e assinaram o Termo de Consentimento Livre e Esclarecido. A identidade dos participantes foi preservada mediante designações alfanuméricas, pois, ao longo do artigo, foram caracterizados como EH (Enfermeiro Hospitalar), TEH (Técnico de Enfermagem Hospitalar), EAB (Enfermeiro de Atenção Básica) e TEAB (Técnico de Enfermagem Atenção Básica), seguidos do número das suas respectivas entrevistas.

### Referencial teórico-metodológico

A Teoria da Complexidade foi utilizada para o processo de interpretação dos dados. A escolha desse referencial se deu por sua capacidade de articular princípios que valorizam a multidimensionalidade dos fenômenos sociais do conhecimento, de modo a sinalizar elementos como a patologia do saber sustentada na fragmentação de informações, bem como na desarticulação do conhecimento com a realidade objetiva^(12)^.

Para o processo analítico dos dados, foi empregada a *Grounded Theory* (GT), em sua vertente construtivista da escola “corbiniana”, cuja abordagem comparativa, amostragem teórica, elaboração e integração de conceitos resultam em três dimensões paradigmáticas, quais sejam: condições, ações-interações e consequências^(13)^.

### Tipo de estudo

Pesquisa qualitativa, do tipo explicativa, que correlacionou as subcategorias em torno de categorias para explicar a realidade do objeto investigado^(14)^. Para a confiabilidade analítica da pesquisa, foram empregadas as diretrizes do Equator, notadamente o guia *COnsolidated criteria for REporting Qualitative research*.

### Procedimentos metodológicos

Os participantes foram caracterizados a partir de um formulário com dados profissionais sobre formação e tempo de trabalho. Para uma aproximação dos participantes com a temática abordada, fora realizada uma dinâmica de sensibilização temática, apoiada na compreensão de que os significados sobre um dado fenômeno devem emergir das mais profundas reflexões sobre a realidade significada^(14)^. Sendo assim, a pesquisadora principal, antes de iniciar a entrevista, disponibilizou dois materiais em formato de artigo contendo informações sobre título, revista, autores, instituições, resumo estruturado e até *link* de acesso. Em seguida, solicitou que os participantes analisassem os materiais com o tempo que considerassem necessário. Após esse processo, a pesquisadora inqueriu o participante sobre a teor de veracidade dos materiais entregues. Cumpre destacar que, em um deles, continha dados totalmente falsos e que foram entregues em formato impresso, e após a dinâmica, foram devidamente recolhidos.

Em seguida, foram realizadas entrevistas semiestruturadas, cujas perguntas indutoras foram: o que você entende por *fake news*? Como você percebe essa realidade no contexto da saúde? Como você percebe *fake news* no contexto da enfermagem? A partir das respostas, perguntas circulares foram elaboradas, de modo a favorecer a apreensão consubstanciada dos dados. As entrevistas aconteceram em encontros individuais, em ambiente reservado, no próprio cenário de estudo, em horários previamente acordados e que não comprometessem as atividades laborais dos entrevistados. As entrevistas foram gravadas em meio digital (áudio), e tiveram duração média de 30 minutos cada. Não foram repetidas com um mesmo participante. Foram empregados memorandos analíticos durante toda a coleta e análise, processo este que ocorre de forma simultânea na GT.

Os memorandos possibilitaram reflexões sobre o desenvolvimento dos conceitos, auxiliando na compreensão da saturação teórica dos dados, ou seja, quando o conceito apresenta densidade teórica, sustentado em suas respectivas subcategorias. O processo de saturação teórica fora discutido entre os pesquisadores antes da coleta de dados ser finalizada.

A coleta de dados ocorreu sob condução de pesquisadora enfermeira com *expertise* no método de coleta de dados e no referencial metodológico adotado. Não houve nenhum conflito de interesse relacionado aos participantes do estudo, tampouco ao cenário da pesquisa.

### Cenário do estudo

Os dados foram coletados em dois cenários: um hospital universitário federal e uma clínica da família situados na cidade do Rio de Janeiro, RJ. O contexto hospitalar apresenta diversas especialdiades clínicas, contendo cerca de 550 leitos. A clínica da família escolhida para o estudo foi inaugurada em dezembro de 2010. A unidade possui 14 equipes de saúde da família, e beneficia 52 mil pessoas, proporcionando 100% de cobertura de saúde da família. A escolha por dois cenários de estudo foi sustentada na hipótese de que o contexto pode influenciar a dinâmica de acesso e disseminação de informações, tanto no que diz respeito ao profissional da enfermagem quanto no que diz respeito ao perfil dos pacientes de cada esfera de atenção à saúde.

### Fonte de dados

Participaram do estudo enfermeiros e técnicos de enfermagem, que compuseram, respectivamente, dois grupos amostrais: profissionais de enfermagem da Atenção Primária à Saúde (clínica da família); e profissionais de enfermagem da Atenção Terciária à Saúde (hospital). Foram incluídos os participantes com, no mínimo, um ano de experiência profissional na instituição, na assistência direta ao paciente, como enfermeiro ou técnico de enfermagem. No cenário hospitalar, os dados foram coletados com profissionais que trabalhavam em unidades de internação adulta (clínica médica e cirúrgica). Foram excluídos os participantes afastados do trabalho, sob qualquer tipo de licença. Os participantes foram convidados presencialmente no cenário do estudo. Não houve recusa ou desistência de nenhum participante.

### Coleta e organização dos dados

A coleta de dados ocorreu entre novembro de 2021 e outubro de 2023. Os dados das entrevistas foram submetidos à análise seguindo as etapas de codificação da GT, que são aberta, axial e integração seletiva. Na codificação aberta, os dados foram segmentados em partes distintas e rigorosamente examinadas, cuja comparação visou captar similaridades entre os dados iniciais (códigos preliminares)^(13)^.

### Análise dos dados

A partir da análise comparativa dos códigos preliminares, houve agrupamento destes, cujo processo permitiu a delimitação de códigos conceituais que foram apresentados como uma representação abstrata de um fato, objeto ou ação que o pesquisador percebe como significativo. O agrupamento dos códigos conceituais por similaridade originou subcategorias, e, dependendo da densidade analítica e conexões entre elas, foram delimitadas as categorias^(13)^. Nesse sentido, a codificação axial exige que o analista já tenha algumas subcategorias construídas para relacioná-las à sua categoria, com vistas a gerar explicações mais completas sobre o fenômeno investigado^(13)^. Na fase de integração, a categoria é desenvolvida a partir das abstrações teóricas de suas subcategorias.

As subcategorias foram ordenadas a partir do modelo paradigmático, que considerou três componentes: condições, que diz respeito os fatores que influenciam o desenvolvimento do fenômeno; ações-interações, que tratam das estratégias de desenvolvimento e enfrentamento do problema; e consequências, que sinalizam as potenciais reações a partir das estratégias implementadas.

## RESULTADOS

Participaram da pesquisa 25 profissionais de enfermagem, dos quais 17 são enfermeiros, sendo 11 do cenário hospitalar e seis oriundos da clínica da família, além de oito técnicos de enfermagem, dos quais seis eram do cenário hospitalar e dois da clínica da família. A média de idade dos profissionais inseridos no contexto hospitalar foi de 35 anos. Já no cenário da clínica da família, a média foi de 28 anos.

O tempo médio como profissionais de enfermagem, no cenário hospitalar, foi de 16 anos para os técnicos de enfermagem e 11 anos para os enfermeiros. Já para os profissionais da clínica da família, a média de experiência profissional foi de dois anos, para os técnicos de enfermagem, e quatro anos, para os enfermeiros. Todos os enfermeiros possuíam, pelo menos, uma pós-graduação. Entre os técnicos de enfermagem, apenas três deles possuíam pós-graduação.

Do processo analítico, emergiu a seguinte categoria: “*Fake news* muito além da dualidade entre verdade e mentira: significados desvelados por profissionais de enfermagem”. A categoria foi estruturada a partir das seguintes subcategorias: “Significados desvelados por profissionais de enfermagem sobre *fake news*”, que no modelo paradigmático assume a conformação de “condições”; “Sistemática do *modus operandi* das *fake news* sob perspectiva dos profissionais de enfermagem”, que trata das ações-interações no modelo paradigmático; “Ciência em questão: a perspectiva sinalizada por profissionais de enfermagem acerca de *fake news* no contexto científico”, que revela as consequências do fenômeno investigado, posicionado no modelo paradigmático. Essa categoria apresenta a lógica não linear sobre *fake news* a partir do campo dos significados desvelados por profissionais da enfermagem.

Cumpre destacar que o desenvolvimento da categoria e de suas respectivas subcategorias resultou do processo de agrupamento de 197 códigos preliminares e 12 códigos conceituais. Portanto, os trechos apresentados são meramente ilustrativos da contextualização dos dados.

### Significados desvelados por profissionais de enfermagem sobre *fake news*

Em alinhamento com o seu título, essa subcategoria revela os significados que enfermeiros e técnicos de enfermagem desvelaram sobre *fake news*. Nessa conjuntura, foi possível identificar que os participantes do estudo compreendem tal realidade, de maneira sistêmica, à medida que conseguem contextualizar a existência de *fake news* para além da ocorrência de informações falsas isoladas.

Embora os participantes do estudo tenham considerado que *fake news* são informações não comprovadas, percebem também que não se limitam à tradução livre do termo (notícia falsa), pois compreendem que há um processo que envolve não somente a dualidade entre verdade e mentira, já que, nas *fake news*, essas características estão intencionalmente implicadas.

Desse modo, para os profissionais de enfermagem, *fake news* conformam processo de elaboração e divulgação de mentiras que assume conteúdo (informações falsas, parcial ou integralmente), forma (mecanismos de elaboração e divulgação, com ênfase na internet, especialmente a partir das mídias sociais) e intencionalidade direcionada ao alcance de objetivos aparentemente definidos (convencimento de um grupo de pessoas/população para tomada de decisão e/ou manutenção de comportamentos).

Para melhor compreensão desse processo, a partir do que sinalizaram os participantes, tem-se, a seguir, trechos destacados que evidenciam a dinâmica de desenvolvimento de *fake news* em forma-conteúdo-intencionalidade.

Forma: o modo como se diz algo, e isto envolve tanto os veículos em que o conteúdo é disseminado quanto os emissores da informação. Os dados, nesse sentido, destacam: o argumento de autoridade (quem diz a informação); a linguagem de fácil acesso para uma compreensão facilitada (como a informação é dita; linguagem); e onde se diz a informação.

Quem diz a informação:

*Por exemplo, a classe médica é uma classe que é supervalorizada na nossa sociedade, e quando um deles fala algo sobre determinado assunto para o paciente, aquilo torna-se um fato incontestável, e isso pode se tornar perigoso.* (EH4)

Como diz:

*Eu percebi que as fake news que circulavam sobre a COVID-19 sempre estavam escritas de uma forma simplória, vamos dizer, que as pessoas entendiam, sabe?! Então, as pessoas acabam compreendendo de forma verdadeira. Porque elas entendem, e para uma doença desconhecida, onde muito pouco se sabia, acaba fazendo sentido para aquela pessoa ou, no mínimo, causava dúvida a ponto dela achar que poderia ser verdade e passar adiante.* (EH3)

Onde diz:

*Fake news são notícias que são espalhadas nas redes sociais de forma errada.* (EAB14)

*São notícias que aparecem nas redes sociais sem que haja uma comprovação.* (TEAB20)

*Eu entendo que são notícias falsas, notícias erradas ou distorcidas ou mentirosas através das redes sociais.* (TEH12)

Conteúdo: embora inespecíficos, os conteúdos, segundo os participantes, são elaborados com dados da realidade (elementos de verdade) que se associam a informações falsas, de modo que a construção de *fake news* assuma características que tangenciam, de modos variados, a verdade; porém, trata-se de uma realidade distorcida intencionalmente.

*Ah, são coisas inventadas! As pessoas pegam uma pequena informação e distorcem sobre aquilo, sobre aquele assunto, sabe?! Assim, não tem provas o suficiente para comprovar aquilo.* (EH02)

*Fake news são todas as informações distorcidas, uma mistura de mentira com verdades* [...] *que vem com algumas verdades misturadas com mentiras.* (EAB13)

*Fake news é tudo que não é verdadeiro. Ou que parcialmente é verdadeiro. Existem fake News. Tem algo ali que pode ser verdadeiro.* (TEH07)

*Acho que notícias que as pessoas pegam um gancho de alguma notícia e aumentam. Não vou dizer que elas inventam. Na maioria delas, elas são distorcidas de alguma forma.* (EH5)

*Acho que notícias que as pessoas pegam um gancho de alguma notícia e aumentam. Não vou dizer que elas inventam, na maioria delas elas são distorcidas de alguma forma.* (TEH05)

*Fake news são notícias mentirosas, sem fundo verídico, né?! É isso, notícias compartilhadas sem comprovação, sem base.* (EAB17)

*As pessoas pegam uma pequena informação e distorcem sobre aquilo, sobre aquele assunto, sabe. Assim, não tem provas o suficiente para provar aquilo.* (EH2)

Intencionalidade: a distorção da informação, conforme descrito acima, está diretamente inserida no processo de construção de *fake news*, porém com a intencionalidade de quem as elabora. Para os profissionais de enfermagem, essas notícias são, portanto, elaboradas intencionalmente para não dizer a verdade. Tal realidade está, segundo eles, inclinada à difamação, calúnia e distorção da realidade.

A intencionalidade observada pelos participantes é de uma conjuntura complexa, pois envolve desde inclinações pessoais, influências da polarização política, até interesses econômicos.

*Na vacinação agora, como tem pessoas que não querem se vacinar, elas acabam influenciando outras com informações que não são verdadeiras.* (EH1)

*Por exemplo, ultimamente com relação à vacina, algumas pessoas propagam informações que contradizem a eficácia da vacina e desestimulando a população a se vacinar. Está muito interligado com a política também, eu acho.* (EH6)

*Eu entendo por fake news toda notícia que seja enganosa, difamatória ou caluniosa de algum fato.* (EH4)

*Por exemplo, agora, estão circulando notícias falsas envolvendo nosso piso salarial. Estão dizendo que os hospitais não vão aderir a essa nova lei. Cada um fala de uma forma diferente e isso vai gerando uma expectativa e ao mesmo tempo uma tensão. E também tem uma questão política, por exemplo, do governo disparando informações que não são verdadeiras para benefício próprio.* (EH8)

Porém, cabe destacar que não houve especificidades de significados apresentados sobre *fake news* em relação aos contextos hospitalar e de Atenção Básica à Saúde, tampouco entre enfermeiros e técnicos de enfermagem.

### Sistemática do *modus operandi* das *fake news* sob perspectiva dos profissionais de enfermagem

Essa subcategoria revela potenciais condições para o sucesso das *fake news*, de acordo com o que pensam os profissionais de enfermagem. Para eles, a operacionalização das *fake news* se dá, especialmente, mediante mídias sociais como veículos de disseminação por influenciarem diretamente a dinâmica de compartilhamento e consumo dessas notícias, tanto pela celeridade quanto pelos algoritmos, para manutenção do pensamento e de comportamentos de grupos polarizados. Trata-se de processo de retroalimentação da divulgação e consumo de *fake news* que influencia: a massificação das notificas; a credulidade das pessoas (pacientes) em relação ao que consomem nas mídias; e a acessibilidade e disseminação das informações, associadas aos grupos polarizados.

*Então, eu vejo que algumas informações que circulam nas redes. Alguns pacientes acreditam, e daí começam a seguir. Até mesmo quando não tinha vacina, tinha gente que não acreditava na doença.* (EH1)

*Eu entendo que são notícias falsas, notícias erradas ou distorcidas ou mentirosas através das redes sociais: Facebook^®^, Instagram^®^, WhatsApp^®^.* (EH12)

*Essas notícias distorcidas normalmente vêm das redes sociais: Facebook^®^ e WhatsApp^®^. Isso faz com que a gente tenha mais facilidade em se deparar com elas porque todo mundo hoje em dia tem acesso a esses meios de comunicação.* (EAB15)

*A facilidade de acesso às redes sociais eu acho que ajuda muito a propagação* [das *fake news*]. *Hoje em dia, todo mundo tem acesso à internet e se comunica com várias pessoas ao mesmo tempo*. (EH23)

*A internet facilita muito o nosso acesso à comunicação. A gente consegue a um clique saber o que acontecer em outro lugar do mundo.* (TEH03)

*São notícias de circulação em massa, principalmente pelas redes sociais e aplicativos tipo WhatsApp^®^* [...] *as pessoas ficam em seus grupinhos e divulgam nesses grupos informações que elas acreditam. Nem sempre é verdadeiro, mas no grupo ninguém questiona.* (EH9)

A amplificação e propagação de *fake news* são, pelos participantes, compreendidas a partir da celeridade com que as informações são divulgadas em decorrência da agilidade permitida pela internet, bem como pela capacidade de alcançar, simultaneamente, múltiplas audiências. Essa amplificação é também favorecida pela ausência ou limitações de estratégias que funcionem como barreiras que permitam fiscalizar/impedir a disseminação de *fake news* em redes sociais.

*Ah, fake news são notícias falsas que são colocadas na internet, né? E aí tomam uma proporção gigantesca.* (EH3)

*Rede social é um negócio que você consegue disparar mensagens para várias pessoas. No WhatsApp^®^, você recebe diariamente essas notícias que não são verdadeiras. Através dele, você consegue espalhar a mensagem muito rápido.* (TEH22)

*Acredito que a facilidade de acesso desses meios, a falta de uma barreira e a rapidez que você consegue disseminar a mensagem facilita a disseminação dessas mensagens.* (EH21)

*Nos últimos tempos, as fake news ganharam força por conta do WhatsApp^®^, né?! É muito rápido que chegam as mensagens. Às vezes, ainda nem saiu no jornal, mas já está nos grupos na rede social.* (TEAB20)

A natureza contínua e incessante das mídias sociais alcança expansão a partir da interatividade que essas mídias permitem (dinâmica algorítmica) para manutenção de audiências. Para os profissionais de enfermagem, o excesso de informações divulgadas nas mídias sociais também é fator que facilita a propagação das notícias enganosas. Portanto, volume, frequência e celeridade são fatores percebidos pelos participantes da pesquisa na dinâmica de divulgação de *fake news.*

*Nas redes sociais, a informação é diária, constante, 24 horas, postando a todo tempo, e todo mundo pode interagir com aquela informação.* (EH23)

*Na internet e nas redes sociais, a informação vem em um turbilhão, o todo tempo, ao mesmo tempo. É uma avalanche de informação ao mesmo tempo. E ao mesmo tempo que você recebe, você consegue compartilhá-las em um segundo.* (EAB18)

Ao considerarem a complexidade envolvida no desenvolvimento de *fake news* (forma, conteúdo, intencionalidade, natureza das mídias a serviço da divulgação dessas notícias), os participantes revelaram, em seus significados, conexões entre *fake news* e o contexto da saúde, notadamente quando percebem que o comportamento de consumo e compartilhamento dessas notícias pelos pacientes podem aumentar vulnerabilidades em decorrência do *déficit* de conhecimentos dessas pessoas em relação às múltiplas questões relacionadas ao processo de saúde-doença, entre outros assuntos correlatos. Portanto, essa realidade, de acordo com os profissionais de enfermagem, encontra conexões com o nível de educação básica formal e educação em saúde da população.

*Eu acho que falta conhecimento da população. Infelizmente, a gente tem uma população que não tem muito estudo, que não entende o processo de saúde-doença, e tudo que vê na internet acredita e acaba disseminando.* (TEH03)

*Eu acredito que muito por falta de conhecimento. Os pacientes, geralmente, são leigos e não sabem a respeito de saúde*. (EH01)

*Nossa população é bem de baixa renda, desconhece sobre os cuidados de saúde, mas, ao mesmo tempo, todos têm WhatsApp^®^, onde sobra notícia falsa.* (EAB17)

Além do conhecimento formal sobre saúde, os sistemas de crenças e valores podem influenciar a dinâmica de consumo e disseminação de *fake news* pela população, segundo os profissionais de enfermagem entrevistados.

*Eu acho que pode ser meio assim: além das pessoas não buscarem mesmo a veracidade da informação, eu acho que também pode ser por falta de aprofundamento, talvez do conhecimento ou até mesmo da força de vontade de verificar se é real ou não aquela informação. Eu acredito que seja mais por isso. As pessoas não querem parar, de fato, para investigar.* (TEAB16)

*No caso da vacina, a gente viu um grande avanço para o enfrentamento de doenças. São milhões em dinheiro investidos. Muitas pessoas, os cientistas, estudaram para aquilo, para a vacina sair. E muitas pessoas, infelizmente, não acreditam, mesmo estando no século XXI, mesmo com provas de que a vacina funciona, e ainda assim tem gente que acredita que não funciona.* (EH02)

*Os idosos possuem já suas próprias crenças. Fica difícil desmistificar tudo que ele acreditou ao longo de anos. Então, eles se mostram resistentes à mudança.* (EH7)

*Aqui, temos usuários muito humildes, pouco letrados, e muitas das vezes eles trazem com eles crenças ou informações que vizinhos ou parentes repassam para eles.* (EH9)

### Ciência em questão: a perspectiva sinalizada por profissionais de enfermagem acerca de *fake news* no contexto científico

Os dados dessa subcategoria foram expressivamente subsidiados pelo processo de sensibilização realizada com os participantes da pesquisa, ocasião em que foram apresentadas pela pesquisadora uma notícia falsa e uma verdadeira, conforme descrito na metodologia do estudo. Quando questionados sobre como percebem *fake news* na saúde e enfermagem, os participantes sinalizaram que compreendem que essas notícias também alcançam o contexto científico em igual dinâmica (forma, conteúdo e intencionalidade) que percebem no dia a dia.

Para os profissionais de enfermagem, a ciência e os cientistas representam um argumento de autoridade sobre o que é divulgado por eles, e essa representação arraigada socialmente influencia a credibilidade dessas notícias. Todavia, os dados revelaram a importância da proficiência científica dos profissionais para conseguirem identificar *fake news* inseridas nos contextos científicos, conforme podem demonstrar os trechos de depoimentos seguintes:

*São mensagens que são veiculadas através de rede social ou até mesmo em um artigo falso.* (EH10)

*É uma notícia disfarçada de verdadeira, como, por exemplo, um artigo* [...] *por ser artigo científico, a gente é levado a crer, mas foi publicada sem ter passado por validação.* (EH6)

*Então, a informação - uma parte pode não ser verdadeira, mas outra parte dela é* [...] *se você chegar pra mim com uma fala bem convincente, talvez acabe me convencendo porque ela fez faculdade, aí eu vou acreditar.* (EH7)

*Fake news é quando circula alguma informação por rede social, ou divulgam um artigo tido como científico com informações falsas e são propagadas na rede.* (EAB18)

*A cobertura vacinal caiu bastante desde a pandemia, e acho que, desde a década de 90, começou com um movimento antivacina bem grande, inclusive por conta de um artigo que foi mal feito e lançado em uma revista famosa e tudo.* (EH19)

*Teve uma revista super bem conceituada que chegou a publicar um artigo importante. Eu acho que falando da COVID-19. Depois, eles foram ver que aquele artigo não era verdadeiro. Aí a própria revista teve que dar uma nota que aquele artigo não era verdadeiro. Mas assim, fica muito complicado para nós, uma revista importante, você pegar ela e ler um artigo e falar: “Eu acho que isso não é verdadeiro!”.* (EH23)

*Quando é um artigo científico, já é algo comprovado, mais sério. A pessoa que escreveu dedicou-se um tempo para estudar aquilo e acredito que isso é verídico* [...] *verificando se há outras pessoas que falam do mesmo assunto em outro lugar. Não se basear apenas no que uma pessoa está dizendo.* (EH21)

Os participantes do estudo reforçaram a importância de se compreender *fake news* como fenômeno multifacetado, cuja dinâmica de produção e divulgação vai além da simples identificação de *fake news* ou de um contexto de senso comum, alcançando também o cenário da ciência. As representações implicam que *fake news* têm a devida autoridade de argumento (supostamente científicas) e argumento de autoridade (por ser o cientista quem diz a notícia).

## DISCUSSÃO

A complexidade envolvida no desenvolvimento de *fake news* está relacionada ao comportamento de acesso, consumo e disseminação de informações falsas, sejam elas construídas intencionalmente ou não^(15)^. Nessa conjuntura, a patologia do saber, assim designada na Teoria da Complexidade, por apresentar a descontextualização do conhecimento^(13)^, assume, no âmbito das *fake news*, objetivo de atender a propósitos de manipulação coletiva, ou simplesmente porque se trata de informação que se torna obsoleta com o tempo, como ocorre com resultados de pesquisas defasados.

Tais complexidades, associadas ao *déficit* de políticas regulatórias para o tráfego de informações na internet, às polarizações político-partidárias e às fragilidades do conhecimento para um pensamento crítico que estabeleça possibilidades dialéticas de enfrentamento da realidade por meio de recursos e estratégias que permitam critérios qualificadores de verdade, podem fomentar a desregulagem entre a disseminação de *fake news* ^(1)^ e, por conseguinte, a desvalorização do conhecimento científico.

Em uma perspectiva linear, a compreensão sobre *fake news* pode sugerir a tradução livre da expressão, de modo a sinalizar a compreensão reducionista para “notícia falsa”. Em assim procedendo, poder-se-ia inferir que os participantes pensam *fake news* como uma notícia relatada por um indivíduo que conta uma história, cuja interpretação desta informação fica a critério de seu emissor. No entanto, a literatura reitera que a produção da *fake news* pode ser realizada de forma consciente, ou não, a partir dos sistemas de crenças, valores ou até mesmo aspectos cognitivos^(2,7)^.

Os profissionais de enfermagem desta pesquisa significam *fake news* como fenômeno multifacetado, não apenas polissêmico. Nessa conjuntura, *fake news* são compreendidas como informações deliberadamente falsas ou enganosas, com intencionalidade, forma e dinâmica que encontram espaços facilitados em lacunas relacionadas ao *déficit* de comprovação científica ou jornalística a partir de notícias vinculadas na internet. Sendo assim, reitera-se o entendimento de que esta temática não deve ser analisada por uma concepção reducionista^(16)^. Todavia, a ampliação dos significados sobre *fake news* corrobora o entendimento que propõe englobar não só textos deliberadamente falsos, publicados nas redes sociais, mas também vídeos e imagens compartilhadas^(17)^.

Cabe destacar, porém, que, de acordo com os profissionais de enfermagem investigados, esse fenômeno está concentrado no contexto da internet, em especial nas mídias sociais. Esses dados são corroborados pelo fato de que as *fake news* têm, em sua essência, a missão de notícias manipuladas para parecerem reais e o poder viral que estabelecem a partir da forma como são elaboradas, bem como sua disseminação na internet, por meio das redes sociais^(18)^.

A internet e as mídias sociais são meios de comunicação de fácil acesso, cujo consumo de informações tem aumentado exponencialmente nos últimos anos^(2)^. Ademais, tem-se que mídia social significa a sociedade no mundo digital, contexto onde os indivíduos se conectam a partir da internet, favorecendo a comunicação, mas nem sempre para um pensamento plural, visto que são influenciadas por algoritmos que favorecem a manutenção de grupos afins.

Nessas plataformas, é permitido que qualquer pessoa, independentemente de sua credibilidade, dissemine informações com o seu poder de propagação^(2,6)^. Desse modo, a realidade envolvida na elaboração e propagação de *fake news* deve alcançar alerta necessário para utilização das mídias sociais, haja vista a capacidade dessas informações influenciarem, de forma perigosa, comportamentos e opiniões públicas, cujo objetivo de enganar, sobretudo na esfera política, está em convencer o leitor, por exemplo, para determinar posições de controle sob uma condição disruptiva da realidade, a partir de disputas de narrativas^(19)^.

O poder de propagação de *fake news* nas redes sociais, segundo revelam os participantes desta pesquisa, está na agilidade e frequência com que essas informações são transmitidas. Isso deve-se, entre outros fatores, ao célere desenvolvimento da Inteligência Artificial (IA), que, através de algoritmos, possuem a capacidade de triangular dados que permitam a manutenção de grupos específicos de pessoas para ideias e projeções de crenças comuns^(20)^. Assim, a IA atua para influenciar trocas de mensagens, sugerindo novas conexões e consumos de informações elaboradas para maiores interatividades com grupos sociais específicos e afins.

Os algoritmos são um agrupamento técnico e abstrato introduzido no sistema de informação, com o objetivo de interceder nas relações e nas notícias que recebemos^(21)^, de modo que esse mecanismo pode oferecer riscos às escolhas e ao processo de tomada de decisão dos usuários. Ademais, tem-se que os algoritmos são um conjunto de códigos matemáticos elaborados para demonstrar uma posição pessoal definindo o *modus operandi* das informações que circulam nas plataformas de mídias sociais^(22)^. Logo, a IA envolvida nas mídias sociais pode interferir no sentimento de liberdade e confiança das pessoas^(23)^. Acrescenta-se ao problema em questão o fato de as plataformas das mídias sociais, por princípio, não apresentarem a preocupação com a verdade, mas com a utilidade em que se busca conhecer e controlar as informações em uma perspectiva de consumo e de poder^(24)^.

Um outro fator apontado pelos participantes desta pesquisa está direcionado ao âmbito da desinformação da sociedade acerca do cuidado em saúde, e essa realidade é mencionada como contexto favorável para propagação de *fake news*. Assim, é possível observar, ao longo da história da humanidade, que a desinformação sempre transcorreu na construção das sociedades, sob uma lógica de retroalimentação para distintas finalidades. Contudo, o que faz da desinformação uma realidade crescente na sociedade contemporânea é a facilidade de conexões em virtude de uma coletividade extremamente virtualizada^(25)^ associada ao distanciamento de um pensamento crítico capaz de questionar as informações acessadas.

Sob um panorama complexo, o teórico Morin^(13)^ enfatiza que o desenvolvimento do conhecimento consiste, a partir da linguagem e do pensamento, em uma tradução ou reconstrução do saber em que estão perpetuados a concepção de mundo, a subjetividade e os princípios da individualidade. Esse paradigma alerta para a importância de se perceber a potencial fragmentação entre mente e corpo, e emoção e razão, elaborando novos conceitos. Diante disso, ao conceituar *fake news* sob o paradigma da complexidade, entende-se que este se constitui em um ecossistema híbrido, plural e retroalimentado pela heterogeneidade social por meio de narrativas interpretativas e dialógicas pertencentes a um determinado contexto onde os indivíduos agem e pensam de acordo com seu paradigma social, o que, de certa forma, converge com o conceito de pós-verdade.

Na pós-verdade, a disseminação de informações falsas vulgariza a mentira e relativiza a verdade. A importância e a credibilidade dos meios tradicionais de informação são diminuídas por julgamentos pessoais. Já os fatos passam a ficar em segundo plano, de modo que, na era da Pós-Modernidade, as verdades, ainda que científicas, são colocadas como incertas, ao passo em que a sociedade pós-moderna está diariamente em constante construção a partir das mídias sociais, com total valorização da crítica, emergindo, assim, as *fake news*.

As *fake news* podem ser compreendidas a partir dos significados apresentados pelos participantes desta pesquisa, mediante o princípio retroativo da complexidade^(13)^, cuja dinâmica rompe a maneira linear de conhecimento, colocando-o em outro nível, em que o produtor e o produto possuem autonomia no processo informativo, desde a fabricação até a divulgação da notícia. Nessa mesma lógica, a partir do princípio da auto-organização^(13)^, pode-se compreender que as informações falsas possuem autonomia na sociedade pós-moderna, cuja dependência pelas mídias sociais se faz presente ao tempo que emergem e se fortalecem as bolhas informativas.

Os dados desta pesquisa apontaram que os profissionais de enfermagem têm dificuldades para identificar uma *fake news*, apenas por ter sido apresentada em formato de artigo científico. Essa conjuntura pode corroborar o entendimento de que, ainda que existam critérios, é possível que *fake news* passem desapercebidas pelo processo editorial e sejam publicadas sob o discurso de autoridade da ciência. Todavia, quando uma *fake news* é identificada em uma produção supostamente científica, coloca-se em questionamento não somente o veículo e atores implicados em sua divulgação, mas também a ciência, ao encontro do pensamento negacionista^(26)^. Ademais, tal qual o princípio complexo da ecologia da ação, uma vez inserida em um sistema de interações dinâmicas e complexas, uma determinada ação não pode mais ser controlada, e suas consequências passam a ser incertas, fora do alcance do seu agente causador^(12)^. Assim, a produção de uma *fake news* camuflada em um artigo científico pode provocar consequências inimagináveis nos processos decisórios de todos aqueles que consideram a informação falsa, sem dela duvidar, para direcionar condutas clínicas e/ou de cuidados.

Somam-se ao exposto as revistas predatórias que, por não apresentarem compromisso com a integridade científica, podem colaborar para disseminação de informações falsas em formatos de artigos científicos. Esses veículos de comunicação violam a integridade ética da ciência ao adotarem contornos de fabricação de dados, plágios e falsificação, ao tempo que dispensam a avaliação por pares, entre outros importantes critérios de integridade científica^(26,27)^.

Nesse contexto, revista predatória publica artigos oferecendo a rapidez no processo de editoração com títulos generalistas. A problemática do avanço dessas publicações predatórias se deve, especialmente, ao produtivismo acadêmico, que prevê a quantidade em detrimento da qualidade, com o objetivo de retroalimentar parte da indústria editorial que não assume compromisso com a integridade ética-científica, o que torna instável o trabalho do pesquisador, bem como a confiabilidade e credibilidade nas divulgações das informações científicas^(28)^.

### Limitações do estudo

As limitações da pesquisa estão relacionadas ao campo epistêmico, posto que a natureza dos participantes inseridos somente no contexto público de saúde pode, por exemplo, conformar significados específicos sobre *fake news* na saúde e enfermagem.

### Contribuições para as áreas da enfermagem, saúde ou políticas públicas

Ao estabelecerem possibilidades diretas de influências com o comportamento humano, especialmente relacionadas à saúde, as *fake news* conformam importante objeto de interesse da saúde pública. Assim, os significados que os profissionais de enfermagem desvelam sobre esse fenômeno, neste estudo, podem contribuir significativamente para a compreensão sobre as condições, ações-interações e consequências de *fake news* na saúde e enfermagem, de modo a permitir, por exemplo, a dinâmica e *modus operandi* dessas notícias, bem como estratégias para enfrenta-las, inclusive no âmbito da ciência, no contexto da assistência e da educação na saúde.

## CONSIDERAÇÕES FINAIS

Os profissionais de enfermagem compreendem *fake news* de forma sistêmica, reconhecendo que estas vão além da simples dualidade entre verdade e mentira. Para esses profissionais, *fake news* envolvem intencionalidade para influenciar comportamentos ou decisões. Ademais, compreendem que a construção de *fake news* se dá através de uma combinação entre forma, conteúdo e intencionalidade, cujas autoridades de argumento e linguagem acessível desempenham papéis cruciais na disseminação e aceitação dessas informações.

A complexidade envolvida nas *fake news*, para os profissionais de enfermagem, está, portanto, na dinâmica axiomática com que essas notícias são elaboradas, divulgadas, consumidas e retroalimentadas. Assim, do contexto da saúde à produção e consumo da ciência, as *fake news* podem influenciar comportamentos perigosos, que colocam em risco não apenas a qualidade do trabalho dos profissionais de enfermagem e de saúde, mas, principalmente, a saúde pública.

## Data Availability

Os dados de pesquisa estão disponíveis no corpo do artigo.
